# Utility of C-reactive Protein-to-Albumin and Uric Acid-to-Albumin Ratios for Assessing Coronary Artery Disease Severity in Acute Coronary Syndrome: A Study From a Major Tertiary Care Center in Northeast India

**DOI:** 10.7759/cureus.87170

**Published:** 2025-07-02

**Authors:** Bijay K Prasad, Bornali Dutta, Farhin Iqbal

**Affiliations:** 1 Cardiology, Gauhati Medical College, Guwahati, IND

**Keywords:** acs, crp/albumin ratio, northeast india, syntax score, uric acid/albumin ratio

## Abstract

Introduction: Inflammation plays a crucial role in the pathophysiology of acute coronary syndrome (ACS). The C-reactive protein-to-albumin ratio (CAR) and uric acid-to-albumin ratio (UAR) are novel markers of systemic inflammation, and their clinical significance has not been elucidated in ACS.

Aim: This study aims to evaluate the association between CAR and UAR with the severity of coronary artery disease (CAD) in patients with ACS undergoing coronary angiography, as assessed by the SYNTAX score (SS).

Materials and methods: This is a hospital-based cross-sectional study conducted from November 2021 to October 2022. A total of 233 patients with ACS were enrolled in the study, who underwent relevant investigations. Coronary angiograms were used to diagnose the presence of CAD, and its severity was assessed using SS. The correlation between UAR and CAR with CAD severity, as measured by SS, was studied and compared in the study population.

Results: The mean age of the study population was 57.17 ± 10.40 years, with average CAR and UAR values of 8.55 ± 11.05 and 1.62 ± 0.47, respectively. Pearson’s correlation analysis showed significant positive correlations between CAR and SS (r = 0.779, p < 0.001) and between UAR and SS (r = 0.823, p < 0.001). Univariable logistic regression analysis indicated that C-reactive protein, serum uric acid, UAR, and CAR were significantly associated with an intermediate to high SS (p < 0.001).

Conclusion: The present study demonstrated a significant association between CAR and UAR with the severity of CAD, as assessed by SS.

## Introduction

Coronary artery disease (CAD) is a leading cause of both morbidity and mortality globally, placing a heavy economic burden on healthcare systems. Acute coronary syndrome (ACS) encompasses various clinical presentations, including ST-segment elevation myocardial infarction (STEMI), non-ST-segment elevation myocardial infarction (NSTEMI), and unstable angina. In India, ACS poses significant challenges as both a major public health issue and a clinical concern [1,2].

The primary mechanisms underlying ACS include the rupture of atherosclerotic plaques, vasospasm, and subsequent platelet adhesion, aggregation, and thrombosis. Inflammation plays a crucial role throughout the stages of atherosclerosis. Elevated levels of C-reactive protein (CRP) have been linked to an increased risk of cardiovascular events in ACS patients [3]. Serum uric acid, a mediator of inflammation, can increase the risk of atherosclerosis by causing endothelial dysfunction and inflammation in the vasculature [4]. Serum albumin plays a role in inhibiting platelet activation and aggregation; therefore, reduced levels may contribute to coronary artery stenosis mediated by platelet activity [5-8]. Reduced serum albumin levels can increase blood viscosity and disrupt endothelial function. In contrast, albumin plays a protective role by inhibiting platelet activation and aggregation, thereby contributing to the prevention of platelet-induced coronary artery stenosis. Low albumin independently has been linked to worse outcomes following ACS and with an increased likelihood of complications.

The CRP-to-albumin ratio (CAR) has emerged as a promising biomarker for assessing the severity of CAD and predicting short-term outcomes in ACS patients undergoing coronary angiography. Several studies have suggested that a higher CAR is linked to more severe CAD and an increased risk of short-term mortality [9,10]. Similarly, the uric acid-to-albumin ratio (UAR) has been explored as a novel marker for mortality in ACS patients, as well as following percutaneous coronary intervention [11-15]. A few studies have also identified UAR as a predictor of CAD severity, as measured by the SYNTAX score (SS) [13,16]. However, to the best of our knowledge, no study has simultaneously evaluated both UAR and the CAR in relation to CAD severity by SS across the full clinical spectrum of ACS, particularly from our country.

Therefore, this study seeks to address this gap by evaluating the clinical relevance of both biomarkers in a broader ACS population, particularly within the resource-limited context of Northeast India, where access to advanced invasive risk stratification tools may be limited.

This article was previously presented as a meeting abstract at the 74th Annual Conference of the Cardiological Society of India (CSI 2022), held from December 8 to 11, 2022, in Chennai, India.

## Materials and methods

This cross-sectional, single-center, observational study was conducted in the Department of Cardiology at our institute over a one-year period, from November 1, 2021, to October 30, 2022. A total of 233 patients were prospectively enrolled from both the outpatient and emergency departments. Total enumeration sampling was employed in this study. Post hoc analysis for sample size revealed sufficient statistical power to detect meaningful differences in UAR and CAR, thereby providing adequate power for the primary analysis (group sizes of 167 and 66, alpha = 0.05, statistical power > 90%).

The study population included adults >18 years of age diagnosed with ACS according to the Fourth Universal Definition of Myocardial Infarction [17] and undergoing coronary angiography. Patients were excluded from the study if they met the following criteria: previous history of CAD who underwent revascularization, chronic kidney disease (eGFR <60 mL/min/1.73 m²), chronic liver disease (either documented cirrhosis or clinical decompensation such as ascites, hepatic encephalopathy, or variceal bleeding), autoimmune disease, malignancy, active infection, or pregnancy. The study was approved by the Institutional Ethics Committee of Gauhati Medical College (approval number: MC/190/2007/PT-II/NOV 2021/TH-14). Participants provided written informed consent after a detailed explanation of the research purpose and assurance of maintaining privacy and confidentiality.

A detailed history, including age, sex, presenting complaints, risk factors, past medical history, and family history, was recorded. All patients underwent complete clinical examination and necessary biochemical evaluation. Serum uric acid, CRP, and serum albumin were measured using the VITROS 5600 automated analyzer (Ortho Clinical Diagnostics, Raritan, NJ, USA). The reference range is 3.5-5.0 g/dl for serum albumin, 3.5-8.5 mg/dl for serum uric acid, and 0-10 mg/L for CRP. UAR was obtained by dividing the serum uric acid level (mg/dL) by the serum albumin level (g/dL), and CAR was obtained by dividing the CRP level (mg/L) by the serum albumin level (g/dL).

All patients underwent coronary angiography using a Philips Allura Clarity Cath-lab machine (Koninklijke Philips N.V., Amsterdam, the Netherlands). CAG was done through both the femoral and radial routes. The CAG was analyzed, and the lesions were quantified in detail. A detailed report was prepared, tabulated, and analyzed. The SS was calculated using software available at http://www.syntaxscore.com. To reduce interobserver variability, each coronary angiogram was independently assessed by two qualified cardiologists who were blinded to the study objectives and clinical details. Based on the SS, patients were categorized into three groups: low-risk (SS ≤ 22), intermediate-risk (SS = 23-32), and high-risk (SS ≥ 33). The relationship between UAR and CAR and CAD severity, as assessed by the SS, was evaluated and compared across the study population.

Statistical analysis

Continuous variables were expressed as mean ± standard deviation. Categorical variables were shown as percentages and numbers. The Kolmogorov-Smirnov test was performed to assess whether the variables were normally distributed.

Comparisons of continuous data were performed using the Student t-test for parametric variables and the Mann-Whitney U-test for nonparametric variables. The Pearson coefficient was used to describe the degree of correlation between parameters. Categorical data were compared with the chi-square test or the Fisher exact test. The correlation between continuous variables was determined using Pearson correlation coefficients. Linear regression analysis was performed to investigate the association between the severity of CAD (as measured by SS) and inflammatory markers. Logistic regression was used to estimate odds ratios for a 1 mg/L increase in CRP, a 1 mg/dL increase in uric acid, and for each unit change in CAR and UAR. Receiver-operating characteristic (ROC) analyses were used to compare the performance power of UAR and CAR for the intermediate to high SS group. The predictive validities were quantified as the area under the ROC curves. The optimal cut-off value was calculated from the point of maximal sensitivity and specificity (Youden’s index).

A p<0.05 was considered statistically significant. Data was analyzed using Microsoft Excel (Microsoft Corp., Redmond, WA, USA) and open-source statistical software Jamovi version 2.3.28 (Jamovi core team and community, Sydney, Australia).

## Results

A total of 233 patients participated in the study. The average age of the participants was 57.17 ± 10.40 years, with 80.3% of the population being male. Out of 233 patients, 108 presented with STEMI (46.3%), 79 presented with NSTEMI (33.9%), and unstable angina was seen in 46 patients (19.8%). Hypertension was the most common risk factor, affecting 48.9% of the patients, followed by diabetes (38.6%) and dyslipidemia (36.9%). The average CRP level was 31.86 ± 37.97 mg/L, while the mean uric acid level was 6.29 ± 1.44 mg/dL. The mean CAR was 8.55 ± 11.05, and the mean UAR was 1.62 ± 0.47 (Table 1).

**Table 1:** Baseline characteristics of our study population LVEF: left ventricular ejection fraction, SBP: systolic blood pressure, FBS: fasting blood sugar, LDL: low-density lipoprotein, HDL: high-density lipoprotein, CRP: C-reactive protein, CAR: C-reactive protein-to-albumin ratio, UAR: uric acid-to-albumin ratio, HbA1c: hemoglobin A1c, SD: standard deviation, SS: SYNTAX score

Parameters	Mean ± SD (%)	SS ≤ 22 (n-167)	SS > 22 (n-66)	p-value
Age	57.17 ± 10.40	56.28 ± 10.79	59.32 ± 9.11	0.046
Sex male (%)	187 (80.3)	130 (77.8)	57 (84)	0.096
Diabetes (%)	90 (38.6)	53 (31.7)	37 (56.1)	<0.001
Hypertension (%)	114 (48.9)	80 (47.9)	34 (51.5)	0.363
Dyslipidemia (%)	86 (36.9)	58 (34.7)	28 (42.4)	0.294
Smoking (%)	77 (33)	52 (31.1)	25 (37.9)	0.202
LVEF	47.60 ± 8.16	47.61 ± 8.37	47.57 ± 7.68	0.84
SBP	132.70 ± 17.85	133.61 ± 18.71	130.49 ± 15.47	0.18
FBS	112.39 ± 46.69	114.45 ± 41.54	107.38 ± 44.51	0.15
HbA1c	6.25 ± 1.41	6.27 ± 1.41	6.19 ± 1.39	0.48
LDL	133.80 ± 41.00	135.20 ± 41.04	130.41 ± 41.0	0.3
HDL	37.55 ± 7.98	37.11 ± 8.00	38.63 ± 7.89	0.09
Triglycerides	215.50 ± 80.09	220.93 ± 81.77	202.34 ± 74.80	0.07
CRP	31.86 ± 37.97	17.37 ± 10.45	66.13 ± 55.15	0.001
Albumin	3.94 ± 0.25	4.04 ± 0.19	3.68 ± 0.19	0.001
Uric acid	6.29 ± 1.44	5.74 ± 0.75	7.61 ± 1.8	0.001
CAR	8.55 ± 11.05	4.46 ± 2.77	18.47 ± 16.22	0.001
UAR	1.62 ± 0.47	1.42 ± 0.21	2.08 ± 0.58	0.001

On coronary angiogram, we found that 91 patients (41.6%) had single vessel disease (SVD), 59 patients (25.3%) had double vessel disease (DVD), 52 patients (22.3%) had triple vessel disease (TVD), and 22 patients (9.4%) had left main disease. Only three patients (1.4%) had non-obstructive CAD (Table 2).

**Table 2:** Vessel involvement across low, intermediate, and high SS groups SVD: single vessel disease, DVD: double vessel disease, TVD: triple vessel disease, CAD: coronary artery disease, SS: SYNTAX score

SS group	SVD	DVD	TVD	Left main involvement	Insignificant CAD	Total
0-22 low SS	94 (56.3)	51 (30.5)	17 (10.2)	3 (1.8)	2 (1.2)	167
23-32 intermediate SS	3 (6.0)	8 (16.0)	30 (60.0)	8 (16.0)	1 (2.0)	50
≥33 high SS	0 (0.0)	0 (0.0)	5 (31.2)	11 (68.8)	0 (0.0)	16
Total	97 (41.6)	59 (25.3)	52 (22.3)	22 (9.4)	3 (1.3)	233

Upon comparing the baseline characteristics between those with SS < 22 and those with SS > 22, we observed that the mean age, history of diabetes, CRP, uric acid, CAR, and UAR were significantly higher in those with SS > 22 (Table 1).

Upon cross-tabulation and performing the chi-squared test, it was observed that 94 (56.3%) of patients in the low SS group had SVD, and two (1.2%) had insignificant CAD. In the intermediate SS group, 30 patients (60%) had TVD, and in the high SS group, 11 patients (68.8%) had left main disease. Intermediate to high SS were associated with a higher number of patients with TVD and were found to be statistically significant (χ2 = 154.13; df = 8; p < 0.001) (Table 2). Pearson’s correlation analysis showed significant positive correlations between CAR and SS (r = 0.779, p < 0.001) and between UAR and SS (r = 0.823, p < 0.001). Univariable logistic regression analysis indicated that CRP, serum uric acid, UAR, and CAR were significantly associated with an intermediate to high SS (p < 0.001) (Table 3).

**Table 3:** Univariate logistic regression analysis of intermediate to high SS with respect to biochemical parameters CRP: C-reactive protein, CAR: C-reactive protein-to-albumin ratio, UAR: uric acid-to-albumin ratio, SS: SYNTAX score, CI: confidence interval

				95% CI		
Variables	SS ≤ 22	SS > 22	Odds ratio	Lower	Upper	p-value
CRP	17.7 (10.5)	66.1 (55.2)	1.17	1.13	1.23	< 0.001
Uric acid	5.7 (0.8)	7.6 (1.8)	5.07	3.15	8.17	< 0.001
CAR	4.5 (2.8)	18.5 (16.2)	1.82	1.54	2.15	< 0.001
UAR	1.4 (0.2)	2.1 (0.6)	1.9	1.66	4.97	< 0.001

**Figure 1:**
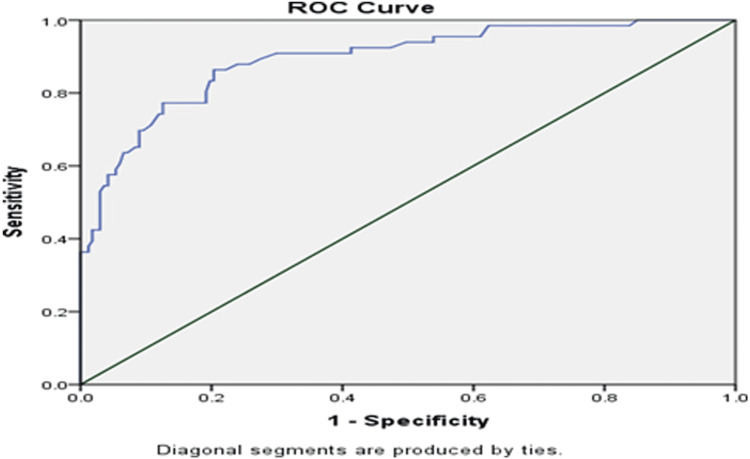
ROC curve for SS and UAR in predicting the severity of CAD in ACS patients with SS > 22 ROC: receiver-operating characteristic, SS: SYNTAX score, UAR: uric acid-to-albumin ratio, CAD: coronary artery disease, ACS: acute coronary syndrome

On analyzing the area under the ROC curve, we found an optimum cut-off value of 1.58 for UAR (AUC: 0.893, 95% CI: 0.84-0.94, p=0.001) with a sensitivity of 86.3% and specificity of 79.6% and a positive and negative predictive value of 62.6% and 93.6%, respectively (Figure 1).

**Figure 2:**
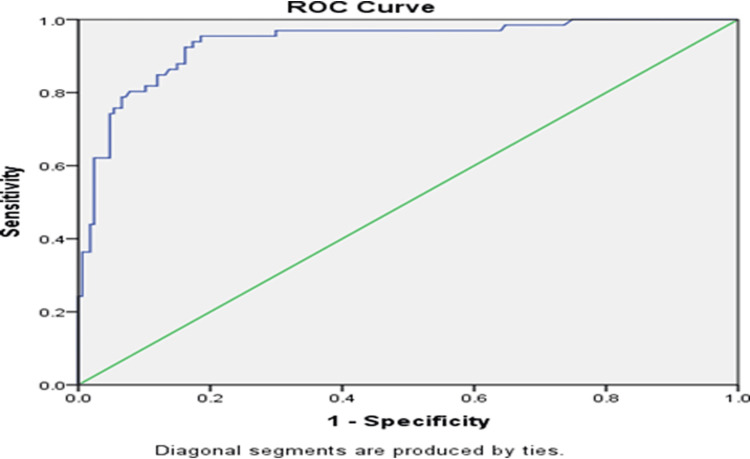
ROC curve for SS and CAR in predicting the severity of CAD in ACS patients with SS > 22 ROC: receiver-operating characteristic, SS: SYNTAX score, UAR: uric acid-to-albumin ratio, CAD: coronary artery disease, ACS: acute coronary syndrome

We also found the ROC curve (AUC: 0.930, 95% CI: 0.90-0.97, p = 0.001) for an optimal cut-off value of 6.14 for CAR, with a sensitivity of 95% and specificity of 81% (Figure 2).

## Discussion

In the present study, we evaluated the utility of CAR and UAR in predicting the severity of CAD using the SS in patients with ACS undergoing coronary angiography at our institute. Our findings suggest that inflammatory markers, including CAR and UAR, are significantly associated with the presence of significant CAD, as indicated by an intermediate or high SS (> 22). Our findings demonstrate that inflammatory markers such as CAR and UAR are significantly higher in patients with more complex and severe CAD.

Our study demonstrated a strong and statistically significant relationship between CAR and SS (r = 0.779, p < 0.001) by Pearson’s correlation analysis. This finding is consistent with the results reported by Cagdas et al. [10], who identified elevated CAR (OR: 1.014; 95% CI: 1.004-1.023; p < 0.001) as an independent predictor of intermediate to high SS in patients with ACS.

Upon analyzing the relationship between UAR and SS, we found a strong correlation between elevated UAR levels and intermediate to high SS. This observation is consistent with the findings of Çakmak et al. [13], who reported significantly higher UAR values in patients with SS greater than 22, indicating a positive association between higher UAR and increased CAD severity. However, an Indian study by Sultana et al. observed no statistically significant difference in UAR among varying severities of CAD [16].

As there were no established cut-off values for UAR, and this cut-off value varied among different studies conducted across various populations in other regions of the world, we analyzed our data using an ROC to identify a cut-off point with optimal predictive values in our study population. We found a strong AUC of 0.893 for UAR in predicting the severity of CAD. According to the ROC curve analysis, at an optimal cut-off value of 1.58, the sensitivity and specificity of UAR in predicting the severity of CAD were found to be 86.3% and 79.64%, respectively, with positive and negative predictive values of 62.6% and 93.6% in our study population. A high negative predictive value suggests that a UAR of < 1.58 was valuable in ruling out severe CAD with an SS of ≤ 22.

Similarly, we conducted an ROC curve analysis for CAR. We identified an optimal cut-off value of 6.14, which demonstrated a sensitivity of 95% and a specificity of 81% in predicting the severity of CAD, defined as an SS of > 22. This finding was statistically significant. In comparison, Çakmak et al. [13] reported an optimal UAR cut-off value of > 0.183 for predicting CAD severity in NSTEMI patients, with a sensitivity of 58.4%, specificity of 81.6%, and an AUC of 0.742. Additionally, Kalyoncuoglu et al. found an optimal CAR cut-off value of 17 for predicting CAD severity in NSTEMI patients, with an AUC of 0.829 (95% CI: 0.770-0.878; p < 0.001) [9]. The variation in optimal cut-off values for CAR and UAR between our study and these previous studies may be attributed to differences in sample size and population characteristics, including geographic variation.

Limitations

This study has several limitations. This was a single-center study with a relatively small sample size, which may limit the generalizability of the findings. As a cross-sectional study, the odds ratios derived from logistic regression represent associations rather than predictive relationships. Blood parameters were measured only once at the time of admission; serial measurements could have provided additional prognostic insights. Furthermore, the study was conducted over a limited period of one year, and we did not assess the association of UAR and CAR with long-term cardiovascular outcomes. The geographic specificity of the study population also limits the broader applicability of the results, making them most relevant to similar regional and clinical contexts. A larger, prospective study with well-defined control groups may help to further validate and build upon our findings.

## Conclusions

To the best of our knowledge, this is the first study to assess the utility of CAR and UAR in predicting the severity of CAD among patients with ACS in Northeast India. Our findings demonstrate that inflammatory markers such as CAR and UAR are significantly higher in patients with more complex and severe CAD. Although this is a preliminary cross-sectional study that cannot establish causality or predictive capability, the observed associations suggest that UAR and CAR may serve as useful, accessible adjunctive markers for assessing CAD severity across the ACS spectrum, especially in resource-constrained settings where advanced imaging or invasive risk stratification tools may not be readily available.
